# Double-Acting Soft Actuator for Soft Robotic Hand: A Bellow Pumping and Contraction Approach

**DOI:** 10.3390/biomimetics7040171

**Published:** 2022-10-20

**Authors:** Hao Liu, Changchun Wu, Senyuan Lin, Yunquan Li, Yonghua Chen

**Affiliations:** 1 The Department of Mechanical Engineering, The University of Hong Kong, Hong Kong 999077, China; 2 The Shien-Ming Wu School of Intelligent Engineering, South China University of Technology, Guangzhou 510641, China

**Keywords:** soft robot applications, multifingered hands, bellow actuation, double acting soft actuator

## Abstract

When compressing a soft bellow, the bellow will contract and pump out the fluid inside the bellow. Utilizing this property, we propose a novel actuation method called compressing bellow actuation (CBA), which can output fluidic power and tendon-driven force simultaneously. Based on the CBA method, a double-acting soft actuator (DASA) combining fluidic elastomer actuator (FEA) and tendon-driven metacarpophalangeal (MCP) joint is proposed for robotic finger design. The proposed DASA exhibits both compliance and adaptiveness of FEAs, and controllability and large output force of the tendon-driven methods. The fluid in the bellow can be either air or water or even integration of the two, thus constituting three different actuation modes. Mathematical modeling of the relationship between bellow compression displacement and DASA’s bending angle is developed. Furthermore, experimental characterizations of DASA’s bending angle and blocking force are conducted at different actuation modes. The double-acting method can availably promote the bending angle of an FEA by up to 155%, and the blocking force by up to 132% when the FEA is water-filled. A soft robotic hand with a forearm prototype based on the DASA fingers is fabricated for the demonstration of finger motion and gripping applications.

## 1. Introduction

Robotic hands and grippers are essential and fundamental components to make the robots interact with the environment. Traditional robotic hands consisted of rigid joints and links [1] and usually have large grasping force, precise control system, and fast movement speed. However, the rigid surface and incompliant interaction make rigid manipulators tend to deform or destroy fragile targets and hard-to-grasp objects with a smooth surface [2]. To overcome these shortcomings, soft robotic hands are good choices to achieve human–robot and robot–environment interaction based on their elastic surface and passive adaptivity [3]. Meanwhile, soft robotic hands can passively catch objects of unknown shapes without complex control algorithms, advanced sensors, and costly precise actuation systems, so the soft prosthetic hands can be safer for amputees [4].

Soft robotic hands are emerging rapidly with various working principles [3], i.e., fluidic elastomer actuator (FEA) [5], tendon-driven actuation [6], variable stiffness actuation [7], adhesion force actuation [8], and smart material (e.g., shape memory alloy [9], shape memory polymer [10], dielectric elastomer actuators [11]), etc. The soft robotic hand proposed in this paper combines the FEA and tendon-driven actuation that are most used in commercial applications and academic studies [6,12]. Inspired by the biological musculoskeletal system, the tendon-driven actuation method is commonly used to mimic a human’s finger. The traditional tendon-driven robotic hand is rigid but underactuated, which means it has more degrees of freedom (DOFs) than its need for operations [13,14]. Tendon-driven actuators are easy to design, fabricate, and control. Meanwhile, tendon-driven robotic hands are usually controlled by motors and have a large grasping force. Although tendon-driven rigid linkage as a robotic finger has a high stiffness to bear a large load, they are not compliant and easy to hurt objects. In addition, because of the advantages of compliance, safety, and adaptiveness, FEA is another widespread soft robot actuation method for robotic hands [15,16]. When the soft chamber is inflated by fluid, the deformation of the FEA is determined by the restraint layer structure wrapped around the chamber. However, FEA fingers are usually too soft to withstand heavy loads. Furthermore, driving high-pressure fluid into the chamber requires a noisy tethered pump system, making it difficult to apply FEAs to portable devices.

**Figure 1:**
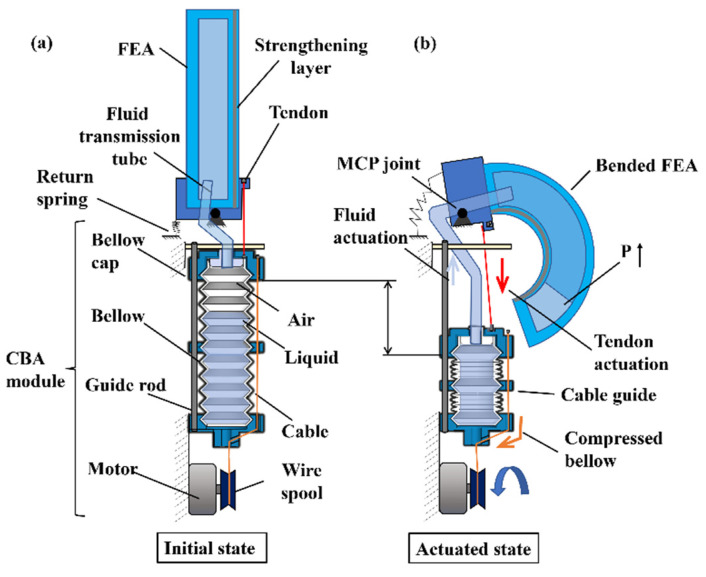
Concept of the DASA. (**a**) The DASA at initial state. (**b**) The DASA at an actuated state by compressing bellow.

Previous researchers have combined two or more actuation modes to design soft robotic hands or grippers for better performance [17,18,19,20]. These studies have proved that the combination of different actuation methods can greatly improve actuator performance such as stiffness, actuation range, output force, motion speed, etc. However, most of these designs required bulky actuation systems for actuation, which also have complex mechatronics designs. To design a portable, controllable, high load capacity, and compliant soft robotic hand, we propose a novel double-acting soft actuator (DASA) design that can pump out fluid and drive a tendon simultaneously with a compact mechanical structure (Figure 1) and concise control system. Inspired by the pneumatic bellow actuators [21], we define the actuation method that can output fluidic power and tendon-driven force simultaneously when squeezing a bellow as compressing bellow actuation (CBA) method. The proposed DASA utilizes the CBA method to achieve double-acting motion. Figure 1 displays the working schematic of the DASA: a tendon and a fluid transmission tube are used to connect the metacarpophalangeal (MCP) joint and FEA finger with the bellow, respectively. The strengthening layer is a rigid woven structure watch band to increase DASA robustness. The contraction of the soft bellow is driven by cables. When cables are pulled by the motor, the bellow will contract along the guide rod, and fluid inside the bellow will be pumped out. Thus, the soft finger is double actuated in two ways: 1. The fluid in the bellow is injected into the FEA, resulting in the pressure increasing in the FEA chamber to bend the FEA. 2. The MCP joint is pulled to rotate by the tendon. The DASA is designed to efficiently achieve the FEA bending and tendon-driven bending simultaneously with an untethered compact actuation system, which keeps the complaint of FEA and increases its bending speed, motion range, and output force through the tendon-driven MCP joint. The novel DASA design has the following merits: 1. The FEA flexion and MCP joint rotation are driven by a single motor, which does not require a complex mechatronic system. 2. The DASA bending speed and flexion angle can be directly controlled by controlling the motor’s rotation speed and rotation angle, which reduced the control system complexity compared to other pneumatic soft actuators driven by a pump system. 3. The DASA design allows different fluids (gas or liquid, or a combination) to be used to satisfy different application requirements. For a combination of air and water, a higher volume ratio of water in the bellow, a larger bending range, higher speed, and larger force could be generated by the finger.

## 2. Materials and Methods

### 2.1. Design and Modeling of the Double-Acting Soft Actuator

#### 2.1.1. Working Principle and Modeling of the Compressing Bellow Actuation

Metal bellows are utilized to absorb the dimensional changes as expansion joints because the changes in temperature and pressure will vary the length and radius of pipes [22,23]. By contrast, bellows made of soft materials can show better shrinkage and elongation than metal bellows. Meanwhile, 3D printing technology makes the soft bellows’ design and fabrication easy without the complicated mold process [24]. Soft bellow actuator and origami actuator can generate linear displacement and fluidic pressure to achieve double-acting motion [25].

**Figure 2:**
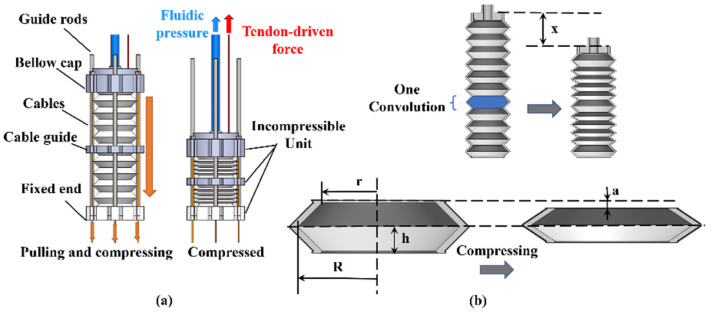
Working principle of the CBA. (**a**) Mechanism of the CBA. (**b**) Geometrical modeling of bellow compression.

The working principle and modeling of the CBA are shown in Figure 2. When a soft bellow is compressed by a bellow cap driven by cables, the fluid inside the bellow chamber will be ejected out and the bellow cap will contract linearly along guide rods. Figure 2b presents the geometrical relationship between the output fluid volume $\Delta V_{Bellow}$ and bellow’s linear displacement x. A bellow consists of many convolutions with heights 2h, and the deformation of one convolution in the compressing process is presented in Figure 2b. Meanwhile, not all convolutions are squeezed when compressing the bellow, such as some convolutions used to connect the device bottom, bellow cap, and cable guide. When the bellow is compressed x, each compressible convolution is compressed 2a=x/ (n−m), where n is the total convolution number and m is the incompressible convolution used to connect other parts. Then, each convolution volume is compressed $\Delta V_{unit}=\left(R^{2}+r^{2}+Rr\right)\left(\pi x\right)/3\left(n-m\right)$. The total volume change is $\Delta V_{Bellow}=\pi x\left(R^{2}+r^{2}+Rr\right)/3$, where the R and r are the inner large and smalle radius of a convolution respectively.

#### 2.1.2. Design and Modeling of the Double-Acting Soft Actuator

**Figure 3:**
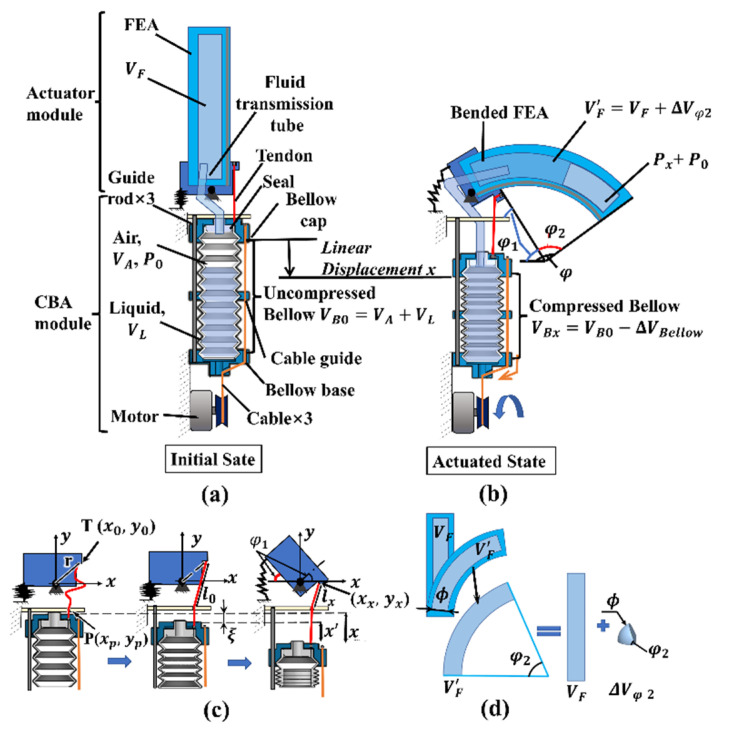
Design and modeling of the DASA. (**a**) Schematic of the DASA. (**b**) The DASA at a bending state. (**c**) Modeling of the tendon-driven MCP joint. (**d**) Modeling of the FEA inner chamber volume change in bending.

Figure 3 shows the schematic diagram of the DASA. The bellow bottom embeds in the bellow base to fix the CBA module, and the top connects to the bellow cap to uniformly bear pull force from three cables that distribute in a circumferential direction. Meanwhile, the bellow cap and cable guide wrapping the bellow are guided by three guide rods with uniform circumferential distribution to keep moving in a vertical direction. To actuate the DASA, a tendon and a fluid transmission tube are used to connect the soft actuator module and the CBA module to transmit fluidic power and tendon-driven force, respectively.

At the initial state displayed in Figure 3a, the initial volume of the uncompressed bellow is defined as $V_{B0}$, which consists of liquid with a volume $V_{L}$ and air with a volume $V_{A}$. The volume of the FEA finger chamber is defined as $V_{F}$. Meanwhile, the pressure inside the bellow and FEA chamber is $P_{0}=P_{atm}$ ($P_{atm}$ is the atmospheric pressure). When the bellow is compressed by a certain displacement x mm, the fluid with a volume $\Delta V_{Bellow}$ inside the bellow will be extruded to the FEA chamber, which leads to the bending of the FEA and MCP joint in Figure 3b. The volume of the compressed bellow is: $V_{Bx}=V_{B0}-\Delta V_{Bellow}$, and the pressure in the bellow and FEA chamber is increased by $P_{x}$ to inflate the FEA. The flexion angle of the DASA is $\varphi =\varphi_{1}+\varphi_{2}$, where $\varphi_{1}$ and $\varphi_{2}$ are the bending angle of the MCP joint and the FEA part, respectively.

Modeling of the relationship between the MCP joint rotation angle $\varphi_{1}$ and the bellow compression displacement x is shown in Figure 3c. The design of the MCP joint can rotate almost 90°, and the tendon displacement used to drive the joint fully rotate is much smaller than the bellow cap displacement utilized to completely compress the bellow. So, we keep the tendon slack at the initial state, and it will be tight after compressing the bellow by a certain displacement ξ, which also means the MCP joint would not rotate when the bellow displacement is less than ξ. When the bellow displacement is larger than ξ, the tendon will be dragged by displacement $x^{\prime }=x-\xi$. To model the bending angle of the MCP joint, the Cartesian coordinate system is established with the joint as the origin as shown in Figure 3c. We define the end of the tendon as point T, and the distance from T to origin is a constant r, $r=\sqrt{{\left(x_{0}\right)}^{2}+{\left(y_{0}\right)}^{2}}$, where *(*$x_{0}$, $y_{0}$) is the T coordinate at the initial state. At the same time, the tendon length from the end to the point P is $l_{0}$. When the bellow is compressed by displacement x (x>ξ), the T coordinate is ($x_{x}$, $y_{x}$), and $x_{x}=\sqrt{r^{2}-{\left(y_{x}\right)}^{2}}$, $l_{x}=\sqrt{{\left(x_{x}-x_{p}\right)}^{2}+{\left(y_{x}-y_{p}\right)}^{2}}$. The relationship between the tendon displacement $x^{\prime }$ and the T coordinate can be expressed as: $x^{\prime }=f\left(y_{x}\right)=l_{0}-\sqrt{{\left(\sqrt{\left(r^{2}-{\left(y_{x}\right)}^{2}\right)}-x_{p}\right)}^{2}+{\left(y_{x}-y_{p}\right)}^{2}}$. The rotation angle of the MCP joint can be calculated by:

(1) $$\varphi_{1}=acrtan\left(\frac{y_{0}}{x_{0}}\right)-acrtan\left(\frac{f^{-1}\left(x^{\prime }\right)}{\sqrt{r^{2}-{\left(f^{-1}\left(x^{\prime }\right)\right)}^{2}}}\right).$$

We use the soft fiber-reinforced bending actuator to design the FEA part and add a strengthening lay to promote finger stiffness. The detailed principle, modeling, and manufacturing process of the fiber-reinforced soft actuator can refer to the website [26]. The FEA volume change $\Delta V_{\varphi_{2}}$ in inflating process has been proofed by previous research as shown in Figure 3d, $\Delta V_{\varphi_{2}}=\frac{\varphi_{2}}{270^{{}^{\circ}}}\pi \phi^{3}$ [20]. For the situation that fluid inside the bellow and FEA is only air, $V_{L}=0$ and $V_{B0}=V_{A}$. According to the general gas equation, we have $P_{0}\left(V_{F}+V_{A}\right)=\left(P_{0}+P_{x}\right)\left(V_{F}^{\prime }+V_{A}^{\prime }\right)$ when the bellow linear displacement is x. We have $V_{F}^{\prime }=V_{F}+\Delta V_{\varphi_{2}}$ and $V_{A}^{\prime }=V_{A}-\Delta V_{Bellow}$. Then, we have $P_{x}=[\frac{\left(V_{F}+V_{A}\right)}{\left(V_{F}+\frac{\varphi_{2}}{270^{{}^{\circ}}}\pi \phi^{3}+V_{A}-\Delta V_{Bellow}\right)}-1]P_{0}$, where $P_{x}$ is the pressure increment in the FEA, and $P_{x}$ is also the function of FEA bending angle $\varphi_{2}$. Referring to paper [15], $P_{x}=\frac{6M_{\theta }\left(\varphi_{2}\right)}{4a^{3}+3\pi a^{2}b}$, $M_{\theta }\left(\varphi_{2}\right)$ is the bending moment due to the stresses acting on the FEA top and bottom layers when the FEA bending angle is $\varphi_{2}$. When the fluid in the bellow is only air, the relationship between the flexion angle $\varphi_{2}$ and the bellow compression displacement x can be expressed as:

(2) $$\varphi_{2}=M_{\theta }^{-1}(\frac{4a^{3}+3\pi a^{2}b}{6}\left[\frac{\left(V_{F}+V_{A}\right)}{\left(V_{F}+\Delta V_{\varphi_{2}}+V_{A}-\Delta V_{Bellow})\right)}-1]P_{0}\right).$$

When the liquid volume inside the bellow $V_{L}>0$, the $V_{B0}=V_{A}+V_{L}$ at the initial state, and Equation (2) still can explain the relationship between the FEA flexion angle $\varphi_{2}$ and the bellow compression displacement x except for the extreme condition, i.e., the bellow, transmission tube, and FEA are almost filled with liquid. The more liquid $V_{L}$ inside the bellow, the less air volume $V_{A}$, and the larger pressure increment $P_{x}$ at the same bellow displacement. When the bellow, transmission tube, and FEA are almost filled with liquid, the primary working fluid is liquid, and Equation (2) cannot be used to calculate the FEA bending angle. Meanwhile, the bellow volume reduction equals the FEA chamber volume increment, $\Delta V_{Bellow}=\Delta V_{\varphi_{2}}$. Then, we can calculate the FEA bending angle when the fluid is only water by:

(3) $$\varphi_{2}=\frac{\left(R^{2}+r^{2}+Rr\right)x}{\phi^{3}}\times 90^{{}^{\circ}}.$$

We can find the relationship between the DASA bending angle φ and bellow compression displacement x:

(4) $$\varphi =\{\begin{array}{c} \varphi_{2},\;\;x<\xi \\ \varphi_{1}+\varphi_{2},\;\;x\geq \xi \end{array}.$$

### 2.2. The Soft Robotic Hand with Five Double-Acting Soft Actuator Fingers

**Figure 4:**
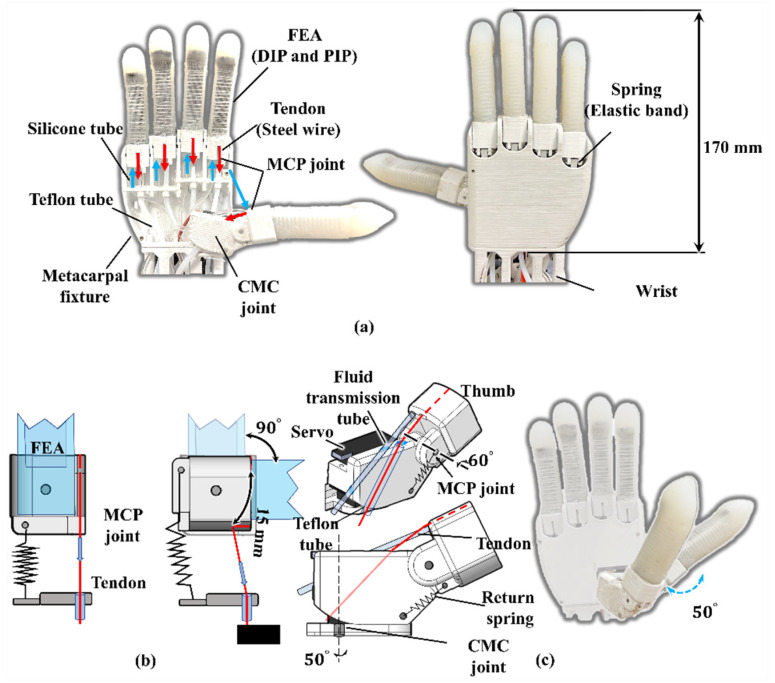
Soft robotic hand with five DASA fingers. (**a**) Soft robotic hand prototype. (**b**) Design parameters of the MCP joints for the four fingers except for the thumb. (**c**) Design parameters of the thumb CMC and MCP joint.

According to the DASA concept, we designed a biomimetic soft robotic hand. Figure 4a demonstrates the prototype of the soft robotic hand. DASA fingers are inserted into a metacarpal fixture serving as anthropomorphic fingers. The FEA part of a DASA finger is molded by silicone rubber (hardness: SHORE 10A), and the bellow is 3D-printed by soft material (eSUN TPE-85A). The FEA structure on each finger functions as phalanges (proximal and middle phalanges), and interphalangeal joints (PIP and DIP joints) on the human palm. For better contact with objects, the FEA fingertip is shaped into an anthropomorphic fingertip shape. For each DASA finger, a silicone tube and a tendon are pulled from a CBA module going through a guide inside the metacarpal fixture. In this design, we use Teflon tubes to reduce friction on the tendon. Meanwhile, the five fingers are controlled by five CBA modules, respectively, and each one can move independently. The 3D-printed wrist casing is used to wrap the silicone tubes and Teflon tubes.

Figure 4b,c present the mechanisms of the MCP joint of the thumb and the other four fingers, respectively. All the joints, metacarpal fixture, and wrist are 3D-printed with material polylactic acid (PLA). For the four FEA fingers (except the thumb), the MCP joint under the FEA can rotate almost $90^{{}^{\circ}}$, and the spring (elastic band) under the MCP joint connected to the metacarpals fixture can achieve finger automatic return after releasing the bellow. In addition, the human thumb can achieve adduction and abduction motion due to the (carpometacarpal) CMC joint. So, in the robotic hand design, the thumb adduction and abduction motion are provided by a servo shown in Figure 4c. When the CBA pulls the tendon, the thumb MCP joint can be bent $60^{{}^{\circ}}$. Moreover, the servo motor allows the CMC joint to rotate 50 degrees to perform more work like tip pinch gestures.

**Figure 5:**
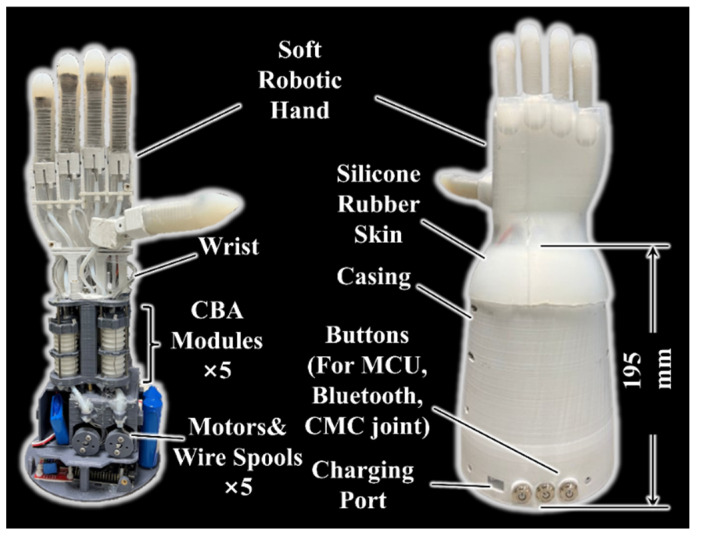
Robotic hand with a forearm prototype.

Based on the soft robotic hand design and DASA concept, we proposed and fabricated a robotic hand module with a forearm displayed in Figure 5. The soft robotic hand actuated by five CBA modules can be easily driven and controlled by motors, which do not need pumps, valves, or any pneumatic or hydraulic components. One CBA module is arranged in the middle of the forearm to control the thumb bending and the other four CBA modules are symmetrically distributed around its circumference to keep the system compact. In addition, five motors (Torque 19 kg·cm) are installed under the CBA module, and each motor controls the bellow compression in one CBA module through three cables connected to the wire spool. Both cables used to compress the bellow and tendon utilized to rotate the MCP joints are steel wires with a diameter of 0.6 mm. The cables’ transmission from motors to CBA modules and tendons’ transmission from CBA modules to MCP joints are guided by Teflon tubes to reduce friction. The control system (battery, Bluetooth module, MCU, etc.), is assembled below the motors, and the prosthetic hand can be controlled by buttons or a smartphone interface. A 3D-printed casing is manufactured to cover actuation and control systems. In addition, a silicone rubber skin covering the hand and wrist is molded to decorate the robotic hand and promote soft contact between the palm and objects. The weight of the robotic hand is almost 1.55 kg, and the height of the forearm casing is 195 mm.

## 3. Results and Discussion

### 3.1. Experimental Characterization of the Double-Acting Soft Actuator

**Figure 6:**
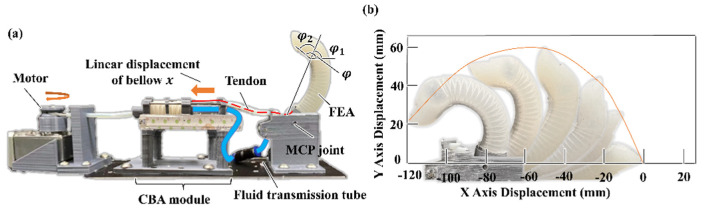
Experimental characterization of the DASA finger. (**a**) Experimental platform. (**b**) Bending trajectory of the DASA finger.

Experimental characterization tests of the DASA finger and the robotic hand are discussed in this chapter. For the bending angle and blocking force experiment of the soft finger, we use the middle finger of the soft robotic hand (the length of the FEA is 80 mm, and the length of the MCP joint is 18 mm). The experimental platform is presented in Figure 6a. The soft bellow has eleven convolutions, and eight convolutions will be evenly compressed during actuation. The other three convolutions connected to the bellow cap, bellow base, and cable guide are not compressed as shown in Figure 2b. For each convolution, the inner large radius R is 10 mm and the inner small radius r is 7 mm. The height of a convolution 2h is 6 mm and the bellow wall thickness is only 1 mm. The length of the FEA inner chamber L is 60 mm. Meanwhile, the chamber radius equals 4.5 mm, and the wall thickness of FEA is 3.5 mm. The bending trajectory of the DASA finger is shown in Figure 6b when it is filled with water. Obviously, the bellow size will affect the pressure increment in the FEA inner chamber. The larger the volume of the bellow, the larger the DASA bending angle.

#### 3.1.1. Bending Angle Test

The first experiment is designed to test the relationship between the compression displacement of the bellow x and the flexion angle of the soft DASA finger φ. $\varphi =\varphi_{1}+\varphi_{2}$, $\varphi_{1}$ is the rotation angle of the MCP joint, and $\varphi_{2}$ is the flexion angle of the FEA part). The fluid in the bellow and chamber can be air, water, or a combination of air and water. When the primary actuation fluid is air, filling the bellow water can reduce initial the air volume in bellow $V_{A}$. The larger the water volume $V_{L}$ in the bellow, the larger the pressure increments after compressing bellow, and larger flexion angle of the FEA. To test how the different water volumes in the bellow affect the bending angle of the FEA part, the experiment of testing the relationship between bellow linear displacement x and the bending angle of the actuator has been conducted in four situations: 1. only air in bellow and FEA (no water), 2. half of the bellow was filled with water, 3. the bellow was full of water, 4. both bellow and FEA inner chamber were full of water. In the fourth condition, the main actuation medium is water, the mathematical modeling is different from the other three situations as shown in Equation (3).

**Figure 7:**
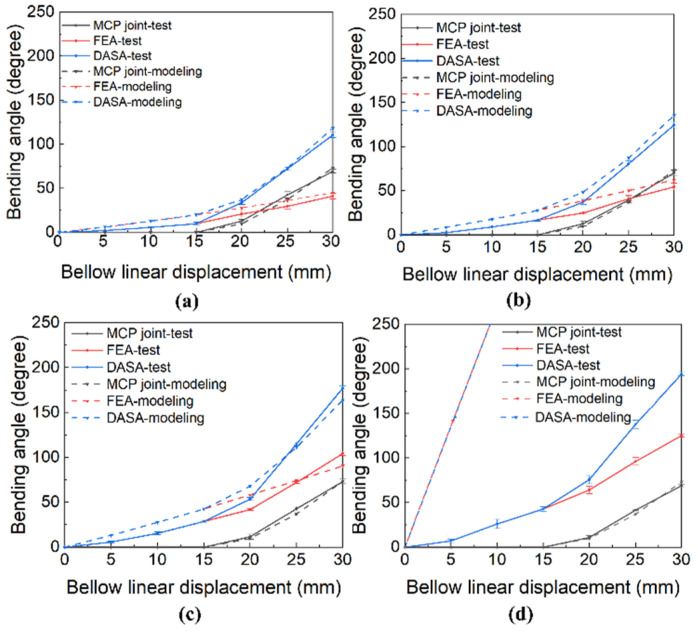
Experimental and modeling results of the finger bending angles vs. bellow linear displacement. (**a**) No water in bellow. (**b**) Half water in bellow. (**c**) Full water in bellow. (**d**) Full water in both FEA and bellow.

For each condition, the bellow was compressed by 30 mm. For every 5 mm displacement increment, we measured the bending angle of the MCP joint $\varphi_{1}$, FEA part $\varphi_{2}$ and the whole DASA finger φ. Each test was conducted five times to reduce experimental error, and the mean values of the tests are plotted in Figure 7 with solid lines. To compare the mathematical modeling with the experimental test, the modeling results are plotted in Figure 7 with dashed lines. As shown in Figure 7, the bending angle increases with the increase in bellow linear displacement, and the more water in the bellow, the larger the FEA flexion angle in the same compression displacement.

The MCP joint bends almost 70 degrees when the bellow is linearly compressed by 30 mm. However, the MCP joint does not rotate when the bellow linear displacement is less than 15 mm. Because of the tendon displacement, we need to find the maximum MCP bending angle, which is far less than the bellow linear displacement that we need to completely squeeze fluid in bellow and out to obtain the largest FEA bending angle; therefore, we make the tendon slack at the initial state like Figure 3c. When the bellow is compressed by displacement ξ (ξ=15 mm), the tendon is tightened and the MCP joint starts to rotate. For Figure 7, the MCP joint rotation angle does not change with the ratio of water volume, which is consistent with the mathematical modeling developed in Section 2.

For the bending angle test of the FEA part with different water volumes, the maximum bending angles are: full water in both bellow and FEA ($124.9^{{}^{\circ}}$) > full water in bellow ($104.2^{{}^{\circ}}$) > half water in bellow ($54.5^{{}^{\circ}}$) > no water in bellow ($41^{{}^{\circ}}$). The bending angle of the FEA increases obviously with the increase in water volume in the bellow. The FEA bends three times more when both bellow and FEA are filled with water compared to the situation when bellow and FEA are filled with gas only. For the modeling of FEA actuated by compressing bellow, the modeling result is close to the experiment result when the FEA is mainly actuated by air. However, for the situation where both the FEA inner chamber and the bellow are almost filled with water, the bending angle modeling result is far larger than the test result. There are reasons caused the huge difference: 1. The pressure increment in the FEA inner chamber is too large (up to 110 kpa), and the silicone rubber cannot deform as the pre-designed result, which means the Kevlar wire wrapped around the inner chamber cannot completely constrain the radial expansion in inflating. 2. It is difficult to achieve full water in bellow, fluid transmission tube, and FEA inner chamber because of the assembly method. Additionally, the residual air can be greatly compressed because of the high pressure generated after fully compressing bellow. Meanwhile, the pressure increment will promote the air solubility of water. 3. The strengthening layer made by the watch band also slightly restricts the FEA deformation.

For the DASA finger, the bending angle equals the sum of the bending angles of the MCP joint and FEA part. When the bellow is fully compressed, the maximum DASA bending angles are: full water in both bellow and FEA ($193.9^{{}^{\circ}}$) > full water in bellow ($177.3^{{}^{\circ}}$) > half water in bellow ($124.7^{{}^{\circ}}$) > no water in bellow ($110.4^{{}^{\circ}}$). The maximum bending angle of the DASA finger is about 1.55 times the maximum bending angle of the FEA when both the bellow and FEA inner chamber are filled with water. Compared with the FEA finger actuated by air ($41^{{}^{\circ}}$), the double-acting method with full water in bellow and FEA promotes the maximum bending angle about 4.73 times ($193.9^{{}^{\circ}}$). The soft bellow compression process is controlled by a motor. The MCP joint makes the DASA finger can bend at a faster speed compared with FEA only in the same motor input. The maximum DASA finger bending angle is $193.9^{{}^{\circ}}$, and it is enough for a robotic hand to pinch and grasp most objects. The DASA finger maximum bending angle can be promoted by increasing the bellow length to fit different work situations. In our previous research, the twisting tube actuation (TTA) method was designed to output the tendon-driven force and fluidic power using a single motor [20]. When the soft finger bends at $190^{{}^{\circ}}$, the TTA method needs a motor input of almost $900^{{}^{\circ}}$, and the CBA method needs a motor input of less than $75^{{}^{\circ}}$ (the bellow is compressed less than 30 mm). So, at the same motor input, the bending angle of the soft actuator based on the CBA method is far larger than the TTA method. Same as the FEA modeling, for the situation where both the FEA inner chamber and the bellow are filled with water, the DASA’s bending angle modeling result is far larger than the test result. The DASA modeling result overlaps the FEA modeling result in Figure 7d.

#### 3.1.2. Blocking Force Test

**Figure 8:**
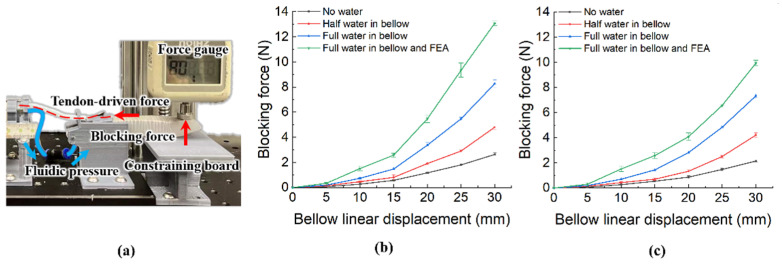
Blocking force test. (**a**) Blocking force test platform. (**b**) Blocking force of the DASA. (**c**) Blocking force of the FEA only.

There are many factors affecting the performance of the robotic hand, such as finger motion range, finger stiffness, complaint contact, etc. The MCP joint greatly improves the bending range of the FEA finger. Previous researchers have proven that the longer FEA inner chamber the larger the bending angle under the same pressure condition [15]. Additionally, tightening the tendon or increasing the bellow’s size can also improve the bending angle of the MCP joint and FEA. However, to design a human-sized robotic hand with a stable system, the parameters mentioned above cannot be too large. We design a strengthening layer inside the FEA to improve the DASA finger’s robustness and stiffness. The strengthening layer makes the soft robotic hand bear heavier loads. The larger blocking force can also help the soft robotic hand grasp objects more stably. We tested the blocking force of the soft finger in the double-acting and FEA-only situations to observe the advantages of DASA in the bending force. The experiment platform is similar to the bending angle test platform in Figure 6a with an extra force gauge and constraining board shown in Figure 8a. The constraining board is set up to constrain the finger motion and the force gauge mounted on the fingertip can measure the blocking force F. In this test, we measured the blocking force of the soft finger in two situations (the DASA finer and the FEA part only). For the two situations tests, the bellow was fully compressed. For every 5 mm bellow linear displacement, we recorded the force gauge value. Meanwhile, we tested the effect of the water volume in bellow and finger on the blocking force in these situations. Each situation was tested five times to reduce errors. The experimental data are plotted in Figure 8b,c, which shows more water in the bellow and FEA inner chamber, the larger the blocking force output in the two situations. The increment of water volume can promote the bending angle and have a higher finger stiffness. The largest blocking force of the DASA finger (13.1 N) is about 1.32 times the FEA-only situation (9.9 N) when both the bellow and FEA inner chamber are filled with water. Because of the rigid MCP joint and the strengthening layer, the largest blocking force (13.1 N) of the DASA finger driven by the CBA method is far larger than the largest blocking force (4.3 N) of the soft actuator driven by the TTA method [20].

#### 3.1.3. Comparison of the Void Volume in Bending

**Figure 9:**
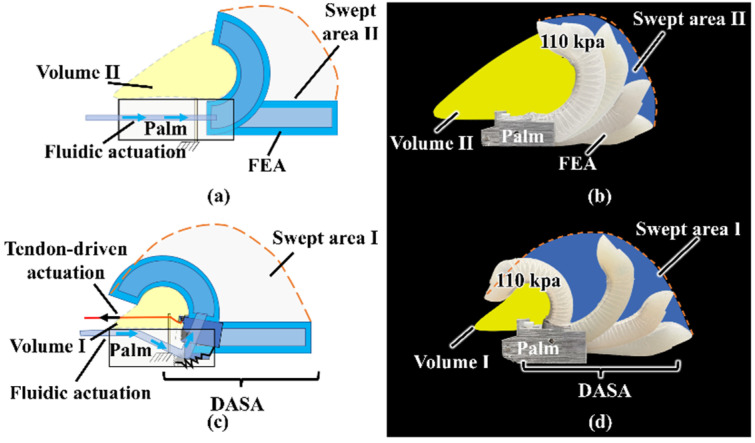
Bending trajectory comparison of the DASA finger and FEA finger. (**a**) FEA finger bending trajectory schematic. (**b**) An FEA finger prototype bending trajectory. (**c**) DASA finger bending trajectory schematic. (**d**) A DASA finger prototype bending trajectory.

When the DASA finger is installed in a humanoid robotic hand, the function of the tendon-driven rotation joint is the same as the metacarpophalangeal (MCP) joint of a human finger, and the FEA part functions as phalanges (proximal and middle phalanges) and interphalangeal joints (PIP and DIP joints) [27]. Figure 9 presents the bending trajectory of the DASA finger and FEA finger when the bellow, transmission tube, and FEA are almost filled with liquid. In Figure 9a,b, the maximum bending angle of the FEA is almost 125 degrees when the bellow is compressed by 30 mm for both the bellow and FEA inner chamber filled with water. The FEA inner chamber pressure is almost 110 kpa when the bellow is fully compressed. The DASA finger can bend up to almost 200 degrees (Figure 9c,d). The FEA finger swept area I in the bending process and the void volume I between the soft finger and palm are shown in Figure 9a,b. The MCP joint actuated by the double-acting method greatly improves the finger-swept area compared with the normal FEA finger shown in Figure 9c,d. The DASA finger has a smaller void volume, which means the robotic hand can grasp objects more tightly, catch smaller targets, and bear a larger load. In addition, the MCP joint greatly improves the bending range of the FEA finger and keeps the compliance of the FEA, because it almost does not contact objects. The MCP joint makes the bending motion of the soft finger more like a human finger. The compressing bellow method easily achieves the double-acting motion without a complicated mechatronic system.

### 3.2. Experimental Characterization of the Untethered Soft Robotic Hand

**Figure 10:**
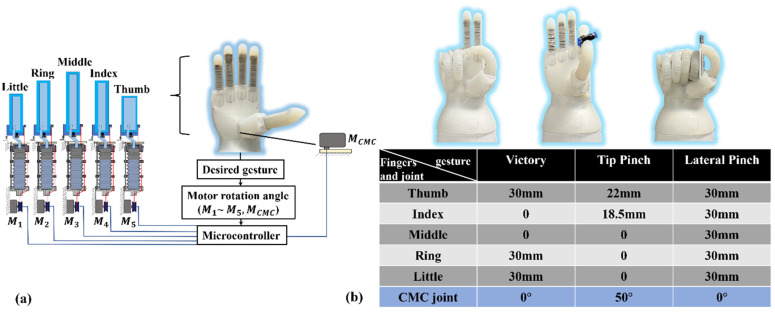
Actuation of the soft robotic hand. (**a**) Actuation strategy. (**b**) Three different hand gestures with related compressed bellow displacement of the CBA module and CMC joint rotation angle.

The compression of the bellow is actuated by three cables which are controlled by a motor. The lengths of the five fingers’ inner chambers are different, so the bending angle of each finger is different when motors rotate at the same angle. The relationship between the flexion angle of the DASA finger and bellow compression displacement has been inferred in Section 2. When the desired gesture is known, each soft actuator’s flexion angle can be known, and the input angle of each motor can be calculated. In addition, the status of the CMC joint is also determined by the gesture directly. Control signals will be transmitted to the six motors to control the bending angle of five fingers and the rotation status of the CMC joint (Figure 10a). Three hand poses were tested to demonstrate the robotic hand control displayed in Figure 10b, i.e., a victory gesture, a tip pinch gesture, and a lateral pinch gesture. The bellow compression displacement for each finger and the status of the CMC joint are shown in Figure 10b.

**Figure 11:**
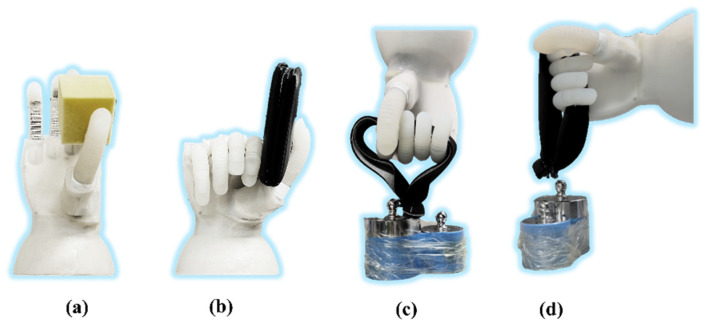
Gripping test. (**a**) Gripping soft object. (**b**) Gripping flat object. (**c**) Hook grip in the horizontal direction. (**d**) Hook grip in the vertical direction.

Figure 11 presents the grasping demonstration and load capability tests of the soft robotic hand. Different from the traditional FEA fingers controlled by a pneumatic system, the soft actuator based on the double-acting method is driven directly by a motor, which means the double-acting finger can start and brake at any position precisely. The precise bending angle controllability and speed controllability of the DASA are shown in the Supplementary Video S1. Additionally, the DASA keeps the compliance of the soft FEA as shown in Figure 11a,b. To show the load capability of the soft prosthetic hand, we demonstrate hook grip in vertical and horizontal directions as shown in Figure 11c,d. The strengthening layer molded in the FEA greatly promotes the stiffness and robustness of the soft finger in the vertical direction, which can not only make the robotic hand bear a heavier load but also help it to grab items more stably. The soft robotic hand can lift objects weighing up to three kilograms in both horizontal and vertical directions. Because of the strengthening layer, the hook grip in the vertical direction is more stable than in the horizontal direction. The adaptability, robustness, and dexterity of the soft robotic hand are shown in the Supplementary Video S1.

## 4. Conclusions

In this research, we propose a novel actuator design for soft robotic hands, which can output fluidic power and tendon-driven force simultaneously using a single motor, defined as the double-acting soft actuator. For the DASA concept, the MCP joint rotation is tendon-driven, and the FEA is driven by fluidic power. The DASA is achieved by the compressing bellow actuation (CBA) method. The mathematical modeling of the relationship between the bending angle of the double-acting actuator and the linear compression displacement of the bellow is built in this paper. Experimental characterizations of the DASA are conducted at different actuation modes. The MCP joint can effectively promote the flexion angle of the FEA up to 155%, and the blocking force up to 132% when the FEA and bellow are filled with water compared to gas. We fabricated a soft robotic hand with five independently controlled DASA fingers, which can lift items weighing up to 3 kg in both vertical and horizontal directions.

There are some limitations in the current design that can be improved in future research. Firstly, the robotic hand prototype is too bulky to function as a prosthetic hand. The actuation system weight and size can be decreased by optimizing the mechanical structure, improving the control system, and selecting more durable materials. Secondly, the DASA finger bending motion is only achieved by controlling the motor directly without sophisticated sensors and feedback control. In the future, tactile sensors can be embedded in the soft finger to assess the contact tendency of the objects, and more sophisticated functions can be implemented.

## Data Availability

Data are available from the authors upon request.

## Supplementary Materials

The following supporting information can be downloaded at: https://www.mdpi.com/article/10.3390/biomimetics7040171/s1, Video S1: Double-Acting Soft Actuator for Soft Robotic Hand: A Bellow Pumping and Contraction Approach.
